# Supplementary material for: Evaluating a Novel Hydrocolloid Alternative for Yogurt Production: Rheological, Microstructural, and Sensory Properties
Source: Foods. 2025 Jun 25;14(13):2252. doi: 10.3390/foods14132252 (PMC12248833; doi:10.3390/foods14132252)
Supplement: Supplementary file 1 [file foods-14-02252-s001.zip › foods-3688093-supplementary.pdf]

Supplementary Table 1: Changes in acidity (% lactic acid) for all samples[1] during storage period

Parameter	Day	Y	YA	YG	YL	YX	YXL
Acidity (% LA)	1	0.9 ± 0.03	1.0 ± 0.06	0.9 ± 0.03	0.9 ± 0.03	1.0 ± 0.00	1.0 ± 0.00
	5	1.0 ± 0.00	1.0 ± 0.00	0.9 ± 0.03	1.0 ± 0.00	1.0 ± 0.03	0.9 ± 0.03
	8	1.0 ± 0.03	1.0 ± 0.00	1.0 ± 0.06	0.9 ± 0.06	1.1 ± 0.05	1.0 ± 0.00
	14	1.0 ± 0.03	1.1 ± 0.03	1.0 ± 0.03	1.0 ± 0.03	1.0 ± 0.03	1.0 ± 0.00

Values are represented as mean ± standard error of mean.
Means with different lowercase letters within the same row are significantly different ($p < 0.05$).
Means with different uppercase letters within the same column for the same parameter are significantly different ($p < 0.05$).
1: Y- control, YA- Yogurt with agar-agar, YG- Yogurt with gelatin, YL- Yogurt with Lactic acid, YX- Yogurt with Disodium 5-guanylate, YXL- Yogurt with novel gel
LA: Lactic Acid
